# Pink and Orange Tattoo Pigments: Two Occurrences of Squamous Cell Neoplasms

**DOI:** 10.7759/cureus.58998

**Published:** 2024-04-25

**Authors:** Erica R Agnese, Alecia Folkes, Jeffrey Fromowitz, Raj Gulati

**Affiliations:** 1 Medical School, Lake Erie College of Osteopathic Medicine, Elmira, USA; 2 Dermatology, Integrated Dermatology, Boca Raton, USA; 3 Dermatology, Dermatology of Boca, Boca Raton, USA; 4 General Surgery, Lake Erie College of Osteopathic Medicine, Elmira, USA

**Keywords:** carcinogens, non-melanoma skin cancer, tattoo complication, tattoo pigment, squamous cell neoplasm

## Abstract

This case report covers the case of a 56-year-old woman with two separate occurrences of squamous cell neoplasms, one arising in pink tattoo pigment and another arising in orange tattoo pigment. A review of the literature was conducted to evaluate the prevalence of malignancy occurring in tattoos. This is rare and there are a limited number of case reports and no large studies done on this condition. Most malignancies in tattoos occur in red, black, or blue tattoo inks and no cases have been reported thus far of malignancy in pink or orange tattoo pigments. Due to the limited number of cases, more case studies are needed to determine the prevalence, risk, and epidemiology of malignancy arising within tattoos.

## Introduction

Tattooing is the act of placing pigment within the dermal layer of the skin [1-3]. It has become very popularized in the United States with up to 25% of individuals between the ages of 18-50 having tattoos [1,3]. While the Food and Drug Administration (FDA) does monitor for adverse outcomes associated with tattoo pigments via consumer and healthcare provider reports [4], the pigments used in tattoo ink currently do not require pre-market approval before release [5]. The tattoo pigments used were originally intended for industrial use, not for human use [3,5-8]. A number of adverse reactions to tattoos have been reported in the literature and include infections, allergic hypersensitivity reactions, foreign body granuloma, sarcoidosis, pseudoepitheliomatous hyperplasia, verruca, keloids, and malignancy [1,6,7,9].

There are a limited number of malignancies occurring in tattoos reported in the literature. The most common malignancy that is reported in tattoos is squamous neoplasms including keratoacanthoma (KA) and squamous cell carcinoma (SCC) [1,7]. Other malignancies associated with tattoos include melanoma, basal cell carcinoma, and lymphoma [3,6,8,9]. A review literature in 2021 yielded 42 cases of squamous neoplasms occurring within tattoos. Red pigment accounted for 75% of cases, and black and multicolored tattoos accounted for the remaining cases [10]. The case presented below is the first case of squamous neoplasm occurring in pink and orange tattoo pigments.

## Case presentation

A 56-year-old woman presented to the practice with the complaint of an eruptive growth in a multichromatic tattoo that had been placed on her left distal medial leg three months earlier. The lesion was growing in the pink portion of her tattoo, and a shave biopsy was performed to evaluate the lesion further. The pathology report showed a mildly atypical acanthotic and endophytic squamous proliferation with some glassy keratinization that extended to the specimen's base. Some exogenous granular material was noted in the papillary dermal histiocytes, consistent with tattoo pigment. The findings were consistent with a KA, and complete removal was recommended. Mohs micrographic surgery was performed. It was cleared at one stage, and the defect was repaired with a primary closure. There was no recurrence of the lesion.

**Figure 1:**
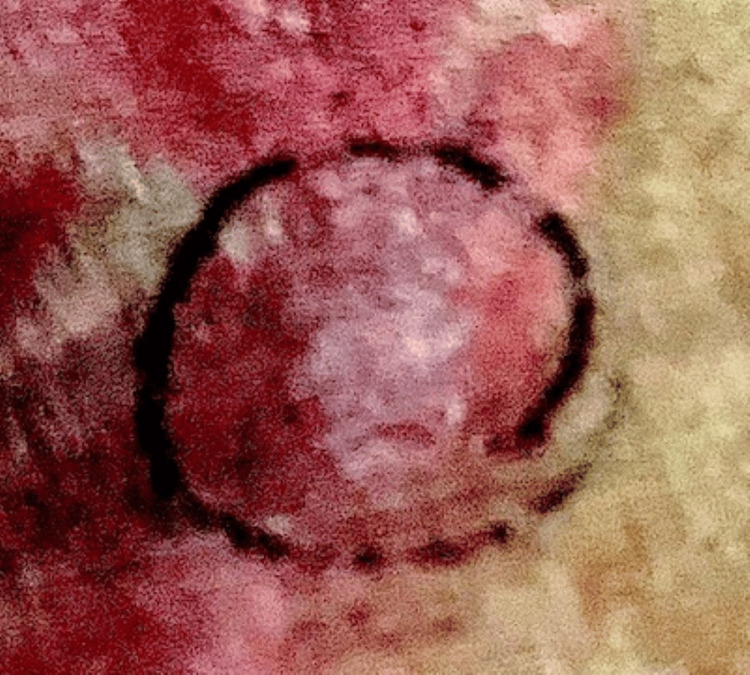
Gross image of the first occurrence of squamous cell neoplasm in pink tattoo pigment. Image Credit: Dr. Jeffrey Fromowitz The lesion is indicated by the black circle

**Figure 2:**
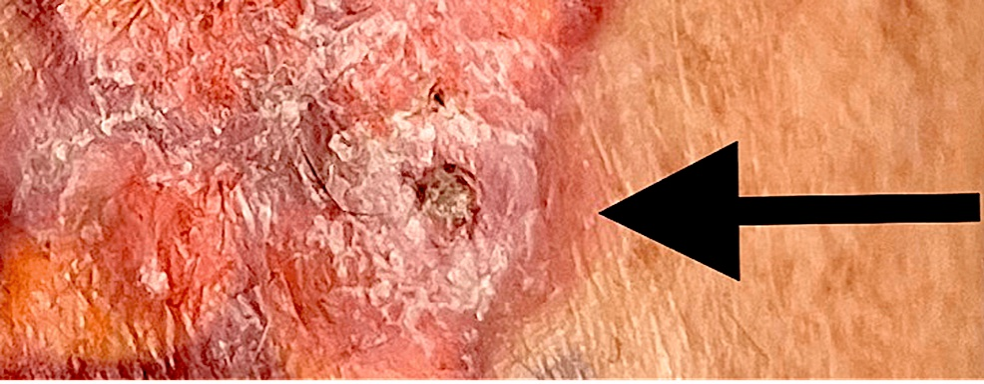
The arrow indicates the second occurrence of squamous cell neoplasm in orange tattoo pigment. Image Credit: Dr. Jeffrey Fromowitz

**Figure 3:**
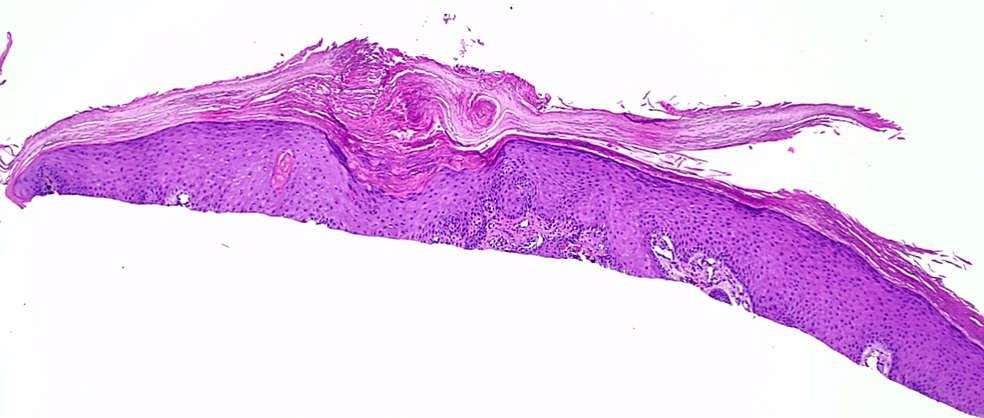
H&E slide of the biopsy specimen of the left distal medial leg at 4x magnification showing mildly atypical acanthotic and endophytic squamous proliferation with some glassy keratinization. Image Credit: Dr. Ivanka Kovalyshyn H&E: hematoxylin and eosin

**Figure 4:**
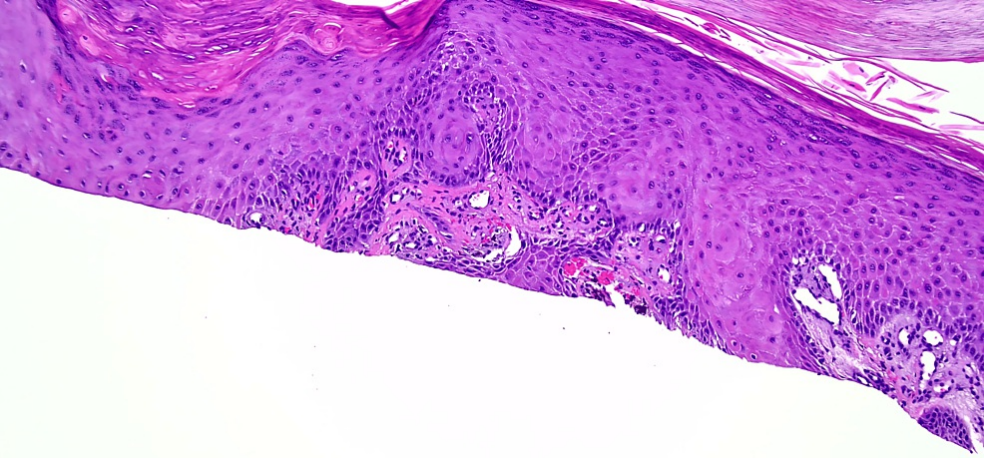
H&E slide of the biopsy specimen of the left distal medial leg at 10x magnification showing squamous proliferation. Image Credit: Dr. Ivanka Kovalyshyn H&E: hematoxylin and eosin

A year following the removal of the initial KA, the patient had the tattoo redone with the additional placements of pink and orange tattoo pigments. Three months following the touch-up, she developed a second eruptive growth in the tattoo, this time in the orange pigment. A shave biopsy was done to evaluate the lesion further. The pathology report showed a well-differentiated SCC with KA features. This SCC was treated with Mohs surgery. It was cleared at one stage, and the defect was repaired with a primary closure. There was no recurrence of the lesion. The patient continues to be followed by dermatology for routine skin exams to assess for the development of further malignancies. Figures 1-2 show gross images of the squamous cell neoplasms in the tattoo and Figures 3-4 show the histological images of the specimen obtained from the left distal medial leg. 

## Discussion

The development of malignancy within tattoos is multifactorial. A study of multiple tattoo inks showed high levels of heavy metals and other carcinogenic compounds, including azo dyes, which are used to make pigmented tattoo inks [7]. The red pigment has the highest association with squamous neoplasms [1,7,9,11], with some studies reporting up to 75% of squamous neoplasms occurring in red tattoo ink [3]. Previously, red ink in the tattooing industry contained high levels of mercury sulfide, which was thought to contribute to carcinogenesis [5,7,9]. A shift has been made toward using organic dyes containing azo pigments, a known carcinogen, and instances of malignancy continue to be reported with the organic dyes [1,5,7,9,11]. In this case, the orange and pink pigments are made from a red base by mixing red with white to obtain pink and red with yellow to make orange. It is unknown if the dyes used in this case were organic or inorganic; however, both types of dyes contain carcinogenic compounds that may have contributed to the development of squamous cell neoplasm in this patient [5,7,9]. 

The trauma of placing the tattoo causes a local inflammatory reaction that triggers an immune response. This is also thought to contribute to the development of neoplasms in tattoos [1-3,6-9,11]. Tattoo-associated malignancies are mostly reported on extremities in areas with high levels of UV exposure [3,8]. The combination of inflammation, carcinogens contained in the tattoo ink, and UV exposure may be synergistic, leading to the eventual development of malignancy [2].

## Conclusions

Malignant neoplasms in tattoos are a rare occurrence, but this may be due to underreporting of incidences. The case study reported here was the first case of malignancy occurring in pink and orange tattoo pigments. The carcinogens found in the red pigment that is used to make the pink and orange inks likely contributed to malignancy development in this patient. This is consistent with the patient's presentation, in which two squamous neoplasms occurred shortly after the tattoo was placed on two separate occasions. Physicians should continue to publish case studies when they encounter this in clinical practice. This will allow for further evaluation of the epidemiology and factors contributing to malignancy in tattoos.
